# Adaptive Shooting Disciplines: A Scoping Review of the Literature with Bibliometric Analysis

**DOI:** 10.3390/healthcare12040463

**Published:** 2024-02-12

**Authors:** Luca Puce, Carlo Biz, Halil Ibrahim Ceylan, Nicola Luigi Bragazzi, Matteo Formica, Khaled Trabelsi, Łukasz Szarpak, Carlo Trompetto, Alessandro Rossin, Nicola Antonio Lanciano, Pietro Ruggieri

**Affiliations:** 1 Department of Neuroscience, Rehabilitation, Ophthalmology, Genetics, Maternal and Child Health (DINOGMI), University of Genoa, 16132 Genoa, Italy; luca1puce@gmail.com (L.P.); ctrompetto@neurologia.unige.it (C.T.); 2 Orthopedics and Orthopedic Oncology, Department of Surgery, Oncology and Gastroenterology (DiSCOG), University of Padua, 35128 Padua, Italy; alessandro.rossin.mc@gmail.com (A.R.); lancianonicola@gmail.com (N.A.L.); pietro.ruggieri@unipd.it (P.R.); 3 Physical Education and Sports Teaching Department, Kazim Karabekir Faculty of Education, Ataturk University, Erzurum 25030, Turkey; halil.ibrahimceylan60@gmail.com; 4 Laboratory for Industrial and Applied Mathematics (LIAM), Department of Mathematics and Statistics, York University, Toronto, ON M3J 1P3, Canada; 5 Istituto di Ricovero e Cura a Carattere Scientifico (IRCCS), Ospedale Policlinico San Martino, 16132 Genoa, Italy; matteo.formica@unige.it; 6 Institut Supérieur du Sport et de l’Éducation Physique de Sfax, University of Sfax, Sfax 3000, Tunisia; trabelsikhaled@gmail.com; 7 Research Laboratory: Education, Motricity, Sport and Health, EM2S, LR19JS01, University of Sfax, Sfax 3000, Tunisia; 8 Department of Clinical Research and Development, LUXMED Group, 02-676 Warsaw, Poland; lukasz.szarpak@gmail.com; 9 Institute of Research Outcomes, Maria Sklodowska-Curie Medical Academy, 03-211 Warsaw, Poland

**Keywords:** para-archery, para-shooting, physiological responses, kinematic assessment, biomechanical assessment, injuries, evidence-based classification

## Abstract

Para-archery and para-shooting, two very popular adaptive shooting disciplines that have earned their place as major official events in the Paralympic Games, share some similarities, as well as distinctive features in terms of rules, physiological requirements, and equipment used. The International Paralympic Committee has a clear responsibility to ensure that all sports within its jurisdiction, including adaptive shooting, can achieve excellence in their respective fields. To achieve this, the conduct of well-designed studies and rigorous research is essential. Although some research has been conducted in this area, a comprehensive and systematic evaluation is still needed. Therefore, the present study aims to provide a thorough review and synthesis of existing research on adaptive shooting disciplines, identify strengths and gaps, and suggest future directions. Arksey and O’Malley’s methodology is leveraged and enhanced with bibliometric and policy analyses to review literature on adaptive shooting. Databases like PubMed/MEDLINE, Scopus, Web of Science, OvidSP, and EMBASE were searched, focusing on studies in adaptive shooting disciplines and analysing these findings through a blend of thematic and statistical methods. Twenty-four studies totalling 483 para-athletes (299 para-shooters and 184 para-archers) are included in this scoping review, focusing on a range of aspects, including physiological responses (n = 9), research design and measurement methods for evidence-based classification (n = 6), biopsychosocial aspects (n = 3), development of new methods and technologies (n = 4), kinematic and biomechanical assessment (n = 1), and epidemiology of injuries (n = 1). Seven articles focused exclusively on para-archery, thirteen exclusively on para-shooting, and four on both para-archery and para-shooting. In conclusion, the available literature on adaptive shooting disciplines is still very limited, especially regarding para-archery compared to para-shooting. This highlights the need for further research in many key areas to ensure a better understanding of the different disciplines and to provide appropriate support for para-athletes. Future research in para-archery and para-shooting should focus on technological innovations, biomechanical studies, and psychological support to enhance athlete performance and accessibility. Addressing the imbalance between the two disciplines, along with injury prevention and global participation, can drive the sports towards greater inclusivity and equity for para-athletes worldwide.

## 1. Introduction

Para-archery and para-shooting are two very popular adaptive shooting disciplines that have earned their place as major official events in the Paralympic Games [1]. Para-archery, with its emphasis on precision and technique, is a beloved sport for people with disabilities to test their accuracy and skill [2]. Since Rome 1960, para-archery has been a long-standing part of the paralympic movement [3], while para-shooting has gained immense popularity among athletes worldwide due to its challenging nature and the required ability to compete at the highest level [4].

Para-shooting is practiced in over 75 countries and has been included in every Paralympic Games since 1976 [4]. The competition format for para-shooting is very similar to that of able-bodied shooting, where athletes aim to hit the target with precision. This requires excellent hand–eye coordination, balance, stability, concentration, patience, and physical and mental strength [5]. The scoring system for adaptive shooting events at the Paralympic and Olympic Games can be difficult to differentiate, and both sports use a functional classification system that promotes fair competition based on athletes’ functional abilities rather than their impairment type [3,4].

In para-shooting, there are three sports classes for athletes with physical disabilities [4]. The SH1 Pistol is for athletes with upper and/or lower limb impairments who compete in pistol events, whereas the SH1 Rifle is for athletes with lower limb impairments who compete in rifle events. The SH2 Rifle is for athletes with upper limb impairments who require assistance with rifle shooting, with or without lower limb impairments. More recently, the paralympic programme included the VI category for athletes with visual impairments, which comprises two 10 m air rifle events: standing and prone. During the competitions, shooters use an audio signal to improve their aim and athletes with visual impairments can request assistance from a sighted guide [6]. 

One of the most common impairments in para-shooting is reduced strength associated with spinal cord injury (tetraplegia or paraplegia or paraparesis), muscular dystrophy, post-polio syndrome, and spina bifida [7,8]. In para-archery, athletes with total or partial absence of bones or joints because of trauma or disease are more common [9]. 

Para-archery is divided into two classes: Open and W1 [3]. Athletes in the “Open Class” have impairments in either the top or bottom half or one side of their body. W1 athletes have impairments in both the top and bottom halves of their body, torso and at least three limbs [10]. 

There are also significant differences in rules between the two sports. Para-archers can use recurve or compound bows, with different rules for each category. In shooting, the rules depend on factors such as the type of gun (air or 0.22 calibre), distance, target, shooting position, number of shots, and time limit [1]. Para-archery requires more upper body strength and endurance, as athletes must hold the bow and draw the string for a longer period [11]. 

A comprehensive study of adaptive shooting disciplines is imperative and is driven by several critical factors. Firstly, from a physical point of view, it is crucial to understand in detail the physiological responses of para-athletes when faced with different levels of physical exertion and unconventional environmental scenarios [8]. It is equally important to analyse their metabolic profile [12,13,14,15] and general athletic performance [16]. Indeed, para-athletes tend to have levels of fitness and brain activity that can differ significantly from those of able-bodied athletes [9,16,17], as well as peculiar and personalised physical responses tailored to the specific nature of their impairment [18,19]. Such a detailed understanding is crucial not only for perfecting their athletic preparation and performance but also for preventing potential injuries [20]. Furthermore, the biomechanical complexities of adaptive shooting are of immense importance. Athletes with disabilities face unique physical challenges that require a tailored approach to equipment design and use [7,21]. A thorough analysis of body mechanics, muscle engagement patterns, and ergonomic considerations is essential [22,23,24,25]. In addition, socio-economic factors have a fundamental impact on the paralympic landscape [26,27]. It is crucial to examine the accessibility of specialised training facilities, the availability of adaptive equipment, and support systems for athletes with disabilities [22]. Disparities in resources among different regions and economic backgrounds can have a profound impact on athletes’ careers and the development of their talent [28]. In addition, research into the long-term effects of competitive activity on athletes’ well-being and overall quality of life is essential [29]. This includes an assessment of the physical and psychological benefits as well as the potential challenges or risks associated with prolonged involvement in sports [2,30]. Finally, classification is a critical component of para-sport as it ensures that athletes compete against others with similar levels of impairment and functional ability [31,32,33]. To ensure the accuracy and fairness of the classification process, it is important to use evidence-based methods that are informed by scientific research [6,34,35,36,37].

Therefore, the research objectives of the present scoping review with bibliometric analysis focus on evaluating the development, trends, and challenges in para-archery and para-shooting, including an assessment of psychological, biomechanical, and socio-economic impacts on athletes. The aim was to provide a comprehensive understanding of these adaptive shooting disciplines, enhance athlete performance and welfare, promote fair and inclusive competition, guide future research and policy development, and strengthen the paralympic movement by highlighting the unique aspects of para-archery and para-shooting within the global sports context.

## 2. Material and Methods

### 2.1. Study Design and Implementation

To conduct the scoping exercise, Arksey and O’Malley’s five-step methodology [38] was fully leveraged, considering subsequent refinements and amendments. The scoping exercise was complemented and enhanced by bibliometric and policy analyses; the aim was not only to map the extant scholarly literature on adaptive shooting disciplines and to assess the scientific community dealing with this topic but also to provide practical implications and recommendations, following the “Patterns–Advances–Gaps–Evidence for Practice–Recommendations” (PAGER) framework developed by Bradbury-Jones and colleagues [39]. 

For the scoping exercise, the authors preliminarily familiarised themselves with the literature, then, a research question was formulated, based on the previously mentioned research aims, following the recommendation of the Joanna Briggs Institute (JBI) and employing the Population (or Participants), Concept, and Context (PCC) mnemonic. On this basis, the inclusion and exclusion criteria were developed: in terms of the study population (or participants), studies were considered eligible if they recruited athletes with any type of disability (including physical, visual, or intellectual impairments) participating in adaptive shooting events. 

The concept of this scoping review revolved around adaptive shooting and related disciplines, encompassing various aspects (psychological, physical, technical, and methodological) ranging from the impact of rules and regulations, techniques, and equipment on performance measures (such as accuracy, scoring, and target distances) to the training and preparation of para-athletes. Finally, the context was related to participation trends, opportunities, and challenges for adaptive shooting, which included the environment and circumstances surrounding adaptive shooting, such as accessibility and inclusion efforts to promote the sport among people with disabilities, the impact and recognition of adaptive shooting in society and within the paralympic movement, or any related policies or issues that affect the participation and development of adaptive shooting.

These inclusion and exclusion criteria guided the process of the identification and selection of relevant scholarly, peer-reviewed studies written in English and designed as original investigations of any type to be included and covered in the present review. To do so, relevant data were abstracted using an Excel spreadsheet, and (either) quantitative or qualitative information was charted, collated, summarised, and reported. Subsequently, a thematic analysis was performed by uncovering the main themes emerging from the body of literature previously identified, dissecting the links and the dynamics underlying these thematic areas, and spotting those that are currently under-developed, in order to inform future research in the field.

### 2.2. Relevant Studies Identification

The search string comprised keywords related to adaptive shooting and related disciplines, connected by the Boolean operator “OR”. More in detail, the search string was as follows: (“handicapped shooting” OR “paralympic shooting” OR para-shooting OR “adaptive shooting” OR “shooting para sport” OR para-shooter OR “shooter with a disability” OR “shooters with disabilities” OR para-archery OR “paralympic archery” OR “amputee archer” OR para-archer OR “archer with a disability” OR “archers with disabilities” OR “disabled archer” OR “adaptive archery”).

Five major electronic, scholarly databases were mined from inception, without language filters/restrictions, namely, PubMed/MEDLINE, Scopus, Web of Science, OvidSP, and EMBASE. Moreover, grey literature (i.e., non-peer-reviewed literature, including theses and dissertations, conference proceedings and abstracts, books, and book chapters, among others) was consulted by mining Google Scholar. The search was conducted up to 1 April 2023, with the screening/selection process carried out independently by two authors (N.L.B. and L.P.). Citations were exported and dealt with by means of the “Research Information Systems” (RIS) file format [40,41]. Duplicate items were removed using open-source citation management software (Zotero version 6.0.30 for Windows, Corporation for Digital Scholarship).

Data extractions were conducted independently by two authors (N.L.B. and L.P.). The following data were extracted: study title and authors, study design, journal, impact factor (related to 2022), Web of Science/Clarivate Ranking, subject areas and categories, sample size and participant features (sex/gender distribution, type of impairment, and para-sports discipline), main study aim, and main research findings. The agreement between the two reviewers was excellent (kappa > 0.90).

### 2.3. Quality Assessment

A quality assessment in scoping reviews is not typically considered mandatory: scoping reviews, unlike systematic reviews, primarily aim to map the extent, range, and nature of research activity in a specific field, as they are used to identify and analyse knowledge gaps, set research agendas, and determine whether a full systematic review is warranted. Being focused on exploring the breadth of a topic rather than assessing the quality of individual studies and often including a wide range of study designs, they usually do not critically appraise the quality of these studies. As such, considering the feasibility, scope, and specific objectives of the present scoping review, quality assessment was not conducted.

### 2.4. Bibliometrics Analysis

VOSviewer version 1.6.18, Gephi version 0.10.1, and Cytoscape version 3.9.1 were utilised for bibliometric data extraction, processing, and graph/network visualisation [40,41]. These graphs/networks were, subsequently, investigated from a quantitative perspective, by calculating an array of various indicators, namely, (i) the count of scholars (authors and co-authors in the bibliographic graph/network); (ii) the number of countries represented in the network; (iii) the number of publications per author or research organisation, both in terms of absolute figures and as a percentage of the total; (iv) the quantity of separate connected components, serving as an indicator of network connectivity; (v) the mean number of adjacent nodes for each node in the network; (vi) the total count of links connecting nodes in the network; (vii) the overall strength of connections between nodes, also known as the total edge weight; (viii) the length of various paths within the network, including the shortest paths (i.e., distance) and the average shortest path length (i.e., characteristic path length), along with other related parameters; (ix) the network’s diameter (the longest shortest path) and radius (the shortest longest path); (x) the density of the network, indicating how connected it is; (xi) the variation in connectivity across the network, often referred to as network heterogeneity; (xii) the extent to which a network is centralised around certain nodes; (xiii) the number of scholar clusters, also known as scholarly communities, within the network; (xiv) the count of research organisation or institution clusters or communities within the network; and (xv) the clustering coefficient, which measures the level of connectivity among a node’s neighbours in the network. Moreover, the number of papers per year was visualised as a time series. 

For further details, the reader is referred to previous publications of the group [40,41].

### 2.5. Statistical Procedures

Descriptive statistical analyses were carried out by synthesising main study features and characteristics, including total sample size, sex/gender distribution, sports discipline representation, and main topics/themes discussed. All analyses were conducted using the commercial software “Statistical Package for Social Sciences” (SPSS version for Windows, version 28, IBM, Armonk, NY, USA).

## 3. Results

### 3.1. Literature Search

The initial literature yielded a pool of 609 items (n = 551 from PubMed/MEDLINE; n = 24 from Scopus; n = 23 from Web of Science; n = 7 from OvidSP; and n = 4 from EMBASE). A total of thirty-five items were duplicated and were, therefore, removed. Five hundred and seventy-four items remained and were screened by title and/or abstract. On the basis of the inclusion/exclusion criteria, 544 items were discarded as being irrelevant. Of the remaining articles, eleven were excluded with reason and are listed in Table 1 (n = 1, lack of sufficient quantitative data; n = 5, focusing on a different population of athletes; n = 2, being review studies; n = 2 being conference abstracts; and n = 1, presenting data that could not be disaggregated and stratified by para-sport discipline) [42,43,44,45,46,47,48,49,50,51,52]. 

A further five articles were retrieved via Google Scholar. Finally, 24 studies were retained for this scoping review [2,6,7,8,9,12,13,14,15,16,17,20,22,23,24,25,27,30,33,35,36,37,53,54]. The included studies focused on a range of aspects including physiological responses (n = 9), research design and measurement methods for evidence-based classification (n = 6), biopsychosocial aspects (n = 3), development of new methods and technologies (n = 4), kinematic and biomechanical assessment (n = 1), and epidemiology of injuries (n = 1). Seven articles focused exclusively on para-archery, thirteen exclusively on para-shooting, and four on both para-archery and para-shooting.

**Figure 1:**
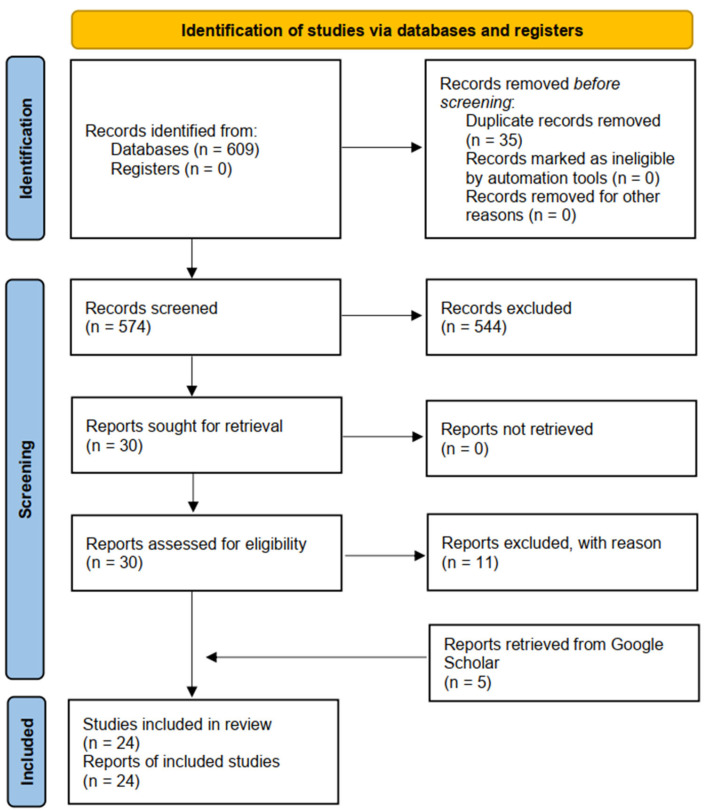
Flowchart adopted in the present scoping review.

The flowchart used in this scoping review is illustrated in Figure 1, and the main characteristics of the included studies are summarised in Table 2 and described in Section 3.2.

### 3.2. Main Study Findings

Physiologically, VO_2peak_ values have been shown to be generally lower in para-shooting and para-archery than in other sports [12,13,14,15]. However, balance levels in these sports are comparable to those in other disciplines [16]. Interestingly, athletes with disabilities such as paraplegia respond differently to hot environmental conditions [8], highlighting the importance of specific training and preparation.

Comparative studies of brain activity during shooting have shown higher attentional demands in para-athletes compared to nondisabled individuals, suggesting the importance of specific mental training [9,17]. Research on spatial perception in visually impaired athletes has identified specific challenges, such as pseudo-negligence, which may affect performance [53].

Regarding the correlation between classification and performance, cross-sectional studies investigating the effect of visual impairment on performance have not found significant correlations, challenging assumptions about the direct influence of visual function on shooting performance [6,33,35,36,53]. Studies evaluating scoring systems in adaptive shooting disciplines demonstrate the complexity and need for continued refinement to ensure fair and unbiased competition [37].

In the area of technological innovation and design, research into equipment design, such as specialised jackets [22], laser target controllers [23], and electromyographic analysis of the muscles involved in the technical gesture [24], highlights the role of technology in improving the performance and comfort of para-athletes. A study evaluating rehabilitation techniques for visually impaired shooters also demonstrated the potential for improved performance through targeted training of visual function [25].

In the biopsychosocial domain, a study examines how social perceptions of disability are influenced by various factors in the context of paralympic sports, highlighting the importance of classification and media representation [27]. Another study presents the inspiring story of a para-athlete who, despite severe physical challenges, has shown remarkable determination and resilience in achieving sporting excellence [30]. Finally, research highlights the physical and perceptual differences between para-athletes and able-bodied archers, highlighting the unique challenges and adaptive abilities of athletes with disabilities [2].

Kinematic studies have shown significant differences in movement patterns, particularly during the shooting phase, among athletes with different disabilities (wheelchair-bound versus standing archers) [7].

Regarding the health and epidemiology of para-athletes, the high incidence of disease in archery, as highlighted by epidemiological studies [20], underlines the importance of health care and preventive measures in these disciplines.

### 3.3. Bibliometric-Based Analysis of the Scientific Output of the Adaptive Shooting Disciplines

**Figure 2:**
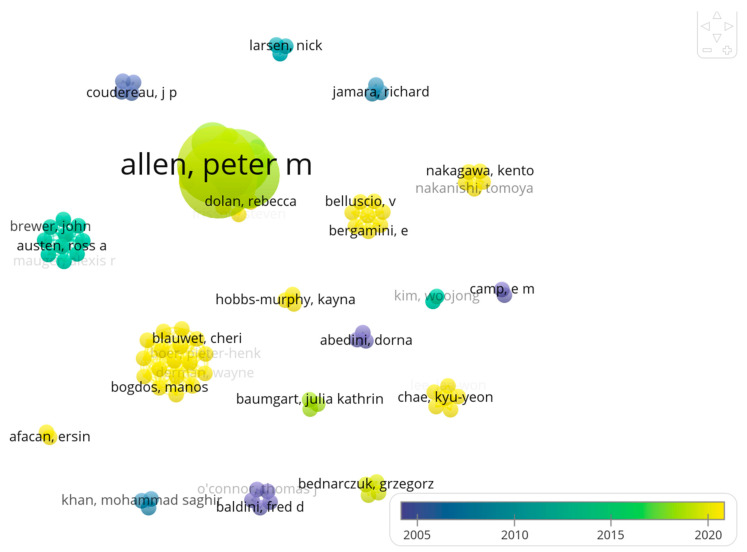
Bibliometric map showing connections of the authors in the field of adaptive shooting disciplines.

The bibliometric analysis is pictorially shown in Figure 2, which illustrates the relationships among authors within the specific research field of adaptive shooting over time. The variously sized and coloured bubbles represent individual researchers, suggesting their level of activity or influence within the field. The larger and more centrally placed the name, the more significant the role that researcher plays in the field, often indicating a higher number of publications or citations. The colours correspond to the time period in which the researchers were most active, as indicated by the timeline at the bottom. This pictorial shows how the field has evolved, with different colours representing different periods. Connections between the bubbles represent co-authorships, indicating collaboration among researchers. 

This analysis allowed the identification of 97 researchers (nodes), of which 17 (17.5%) were highly interconnected. The resulting graph (Figure 2) consisted of 328 links (edges), with a total link strength of 361, and 18 clusters. The most prolific authors were Allen, P.M., and Mann, D.L., each with six items/documents (representing 25.0% of the scientific output reviewed in this scoping review). A list of the four most productive scientists is shown in Table 3. In total, these four authored a quarter of the total literature on adaptive shooting disciplines, with 93.8% of scholars having authored a single document. The main topological features of the scholarly community of authors on adaptive shooting disciplines are shown in Table 4; these features indicate a highly fragmented and dispersed, poorly connected community, characterised by a relatively high number of clusters and a low strength of connections. In terms of years of publication, the first scientific article dates to 1979, with nine articles (37.5%) published since 2020, showing an increasing interest in this para-sport discipline.

The leading institutions were Anglia Ruskin, Cambridge (UK), and Vrije Universiteit Amsterdam, Amsterdam, the Netherlands. In terms of journals, the studies included in our review were published in 20 scholarly journals in the fields of sports science, physiology and experimental physiology, rehabilitation, neuroimaging, public, environmental, and occupational health and sciences, social sciences, economics, and multidisciplinary sciences. 

On the basis of the journal impact factor (related to 2022) and the Web of Science/Clarivate ranking, the top journals were *Medicine & Science in Sport & Exercise*, *Frontiers in Psychology*, *British Journal of Sport Medicine* (Q1 journals), and *International Journal of Environmental Research and Public Health (IJERPH)* (Q1–Q2 journals). Seven articles were published in Q1 journals and another seven in Q2 journals. Two studies appeared in Q3 journals, with a further two articles in Q4 journals. Quartile information was not available for the remaining six journals. In terms of citations, the most cited article was the study by Castle et al. (2013) [8], with 22 citations according to Scopus. Further details are reported in Table 2 and Table 3.

## 4. Discussion

### 4.1. Physiological Responses in Adaptive Shooting Disciplines

Heat acclimatisation has been shown to improve sweat response and thermoregulatory stability in able-bodied athletes, but its effects on athletes with spinal cord injuries are little known. In fact, below the level of a spinal cord injury, the body loses the ability to increase sweat secretion above the baseline rate, resulting in a reduced ability to dissipate heat. As a result, people with spinal cord injuries are at greater risk of developing heat-related illnesses than their able-bodied counterparts [19]. 

To gain a better understanding of the physiological responses to heat acclimatisation in individuals with spinal cord injury, a study was conducted by Castle et al. [8] on five paraplegic athletes participating in para-shooting. The athletes underwent a seven-day heat acclimatisation programme in a hot environment (35 °C and 40% relative humidity) for 90 min per day (20 min arm crank exercise at 50% of VO_2peak_ followed by 40 min rest). Fingertip whole blood was collected at rest on days 1 and 7 to estimate the change in plasma volume. Resting and exercise-induced aural temperature, body mass, and sweat rate were also measured. The results showed that the athletes experienced a decrease in resting aural temperature from 36.3 ± 0.2 °C on day 1 to 36.0 ± 0.2 °C on day 6 (*p* < 0.05). During the heat acclimatisation sessions, the mean aural temperature decreased from 37.2 ± 0.2 °C on day 1 to 36.7 ± 0.3 °C on day 7 (*p* < 0.05). However, no sweat secretion was detected, and no changes in body mass were observed in any of the participants. Despite the lack of sweat response, the athletes had an increase in plasma volume from day 1 to day 7 of 1.5 ± 0.6% (*p* < 0.05). The study suggests that although athletes with spinal cord lesions cannot sweat below the lesion, a repeated hyperthermia programme with limited evaporative heat loss is sufficient to increase plasma volume, probably through changes in fluid-regulating hormones. Finally, improvements in heart rate and perceptual variables were noted. The mean heart rate during the heat acclimatisation sessions decreased from 127 ± 14 beats/min on day 1 to 114 ± 10 beats/min on day 7 (*p* < 0.05), indicating a reduction in cardiovascular stress. Participants also reported a significant reduction in perceived exertion during the heat acclimatisation sessions, meaning that they found the exercise less strenuous. These findings suggest that partial heat acclimatisation can lead to improved cardiovascular function, reduced thermal load, and reduced perceived exertion in athletes with spinal cord lesions. 

The ability to maintain good postural stability enables para-shooters to maintain a stable shooting position, which is essential for accurate aiming and consistent shot placement [55]. Without proper postural stability, the shooter’s movements may cause the aim to deviate, resulting in inaccurate shots and reduced performance. As balance is linked to the ability to correctly perceive the sporting environment by effectively integrating external stimuli, including visual stimuli, visual impairment can lead to a lack of balance and, therefore, poor performance. 

Bednarczuk et al. [16] administered a battery of balance tests to a group of 14 para-shooters, including 4 individuals with congenital disabilities and 10 with acquired disabilities, divided into classes B1, B2, and B3 with four, five, and five members, respectively. Significant differences were observed in the single left leg stance tests, with better balance when standing on one leg with eyes open compared to with eyes closed. Good postural stability may depend on training load, with better results in athletes training more than 5 h per week. No differences were found in the level of dysfunction, although B1 athletes tended to have worse balance in the open-eye tests. Training, therefore, becomes an important tool for improving postural stability by standardising different levels of functionality.

Cortical re-organisation refers to the ability of the brain to adapt and rewire itself in response to changes in the body or environment [56]. Para-athletes who compete in sports that require the use of lower limb prosthetics, such as running, jumping, and sprinting, have been found to exhibit cortical reorganisation in areas of the brain responsible for motor control [9]. Studies have shown that the motor cortex of these athletes is able to adapt to the use of prosthetics and other assistive devices, which may lead to changes in the neural networks that control lower limb movement. 

Nakagawa et al. [9] investigated the cortical re-organisation of lower limb motor representations in an elite archer with congenital amputation of both arms. The athlete had developed a unique technique of using his feet to shoot arrows with great accuracy. The researchers used transcranial magnetic stimulation and functional magnetic resonance imaging to map the cortical motor representations of the athlete’s lower limbs and compared them with those of 12 able-bodied individuals. The authors found several significant findings related to the expansion of lower limb motor representations in the primary motor cortex (M1). First, the study showed that the motor representation of lower limb movements, particularly those involving the toes and knees, was expanded in M1. This suggests that the neural connections associated with these movements have become more developed and efficient. Secondly, the expansion was observed to the lateral side of M1, forming an arc over the representations of trunk and upper limb movements. This suggests that the neural connections responsible for the movements of the lower limbs are closely linked to those of the trunk and upper limbs, and that the brain may integrate these movements more closely than previously thought. Finally, the study identified an enlarged area of corticomotor neurons, which are responsible for the neural control of muscle movement, that innervate the muscles of the lower limb. This suggests that the brain has developed more efficient and sophisticated neural pathways to control lower limb muscle movement.

Kim and Woo [17] used electroencephalography to compare the brain activity of elite shooters, both with and without disabilities, while they performed a visuomotor task. In the study, participants wore standard shooting clothing and equipment, along with a Lycra electrode cap on their head, and fired 20 shots with a compressed air pistol from 10 m. Consistent with the theory of neural plasticity, which suggests that increased activation in extensive cortical regions facilitates functional recovery after spinal cord injury [57], the group with disability showed increased cortical activation. However, despite this difference, the brain regions responsible for visuospatial processing showed comparable levels of activity in both the disabled and nondisabled groups. This is likely because both groups achieved shooting-related neural efficiency through deliberate practice over time. The findings suggest that elite shooters with disabilities may have unique neural processing capabilities that allow them to excel at shooting tasks.

Coudereau et al. [54] conducted a study on the pseudo-neglect effect, which is a phenomenon whereby healthy people tend to have a slight bias towards the left side of space, regardless of their hand [58,59]. This phenomenon was investigated through a comparative study examining the performance in a tactile bisection task between two distinct groups: 10 right-handed archers with visual impairments and 10 nonathletic individuals without visual impairments. The participants were required to estimate the centre of a rectangular wooden block using their sense of touch. The block was presented in different orientations and the participants had to indicate the centre of the longest axis of the block. The researchers measured the accuracy of the participants’ responses and analysed whether they showed a pseudo-negligence effect. The main result was that the para-markers showed a significant pseudo-neglect effect in their tactile accuracy, indicating a leftward bias in their tactile perception. Interestingly, this effect was not observed in non-archers, suggesting that archery training may play a role in the development of this perceptual bias. The authors suggest that the repetitive use of bow and arrow in archery may improve the use of the left hand and arm, leading to a stronger representation of the left side of the body in the brain. This, in turn, could lead to a leftward bias in tactile perception, as seen in the pseudo-neglect effect. 

VO_2peak_ is an important measure of aerobic and endurance capacity and is often used to assess the physical performance of athletes, including those with disabilities in para-sports [60].

Baumgart et al. [12] conducted a systematic review and meta-analysis to investigate peak oxygen uptake in various seated para-sports. The authors also examined the relationship between peak oxygen uptake and various factors, such as age, gender, disability, and fitness status. The study found that peak oxygen uptake normalised for body mass was lower in adaptive shooting than in other seated para-sports. Specifically, from data extracted from three studies [13,14,15], peak oxygen uptake ranged from 15 to 22 mL/kg/min. In sports such as Nordic sitting skiing, values were reported to be more than twice as high (35–52 mL/kg/min) as in adaptive shooting disciplines. Furthermore, peak oxygen consumption in adaptive shooting was influenced by various factors such as age, gender, and level of disability, which is consistent with findings from other para-sports.

In conclusion, adaptive shooting disciplines require less aerobic capacity than other sports. This is because both para-archery and para-shooting are precision sports that rely heavily on accuracy and stability rather than high levels of physical exertion. Consequently, energy requirements are lower, and athletes may not require the same level of cardiovascular fitness and aerobic capacity as athletes in other disciplines. However, the training undertaken by adaptive shooting athletes can lead to significant physiological responses and adaptations that benefit both their specific discipline and general physical well-being in everyday life.

### 4.2. Research Design and Measurement Methods for Evidence-Based Classification in Adaptive Shooting Disciplines

Different sports have different approaches to classifying visually impaired athletes. For example, sports such as swimming divide athletes into three classes based on the World Health Organisation (WHO)’s definitions of low vision and blindness, while sports such as para-shooting group all athletes into a single class. However, the latter approach may give athletes with residual vision an unfair advantage over those who are totally blind. 

To determine whether para-shooters should compete in a single class or separate classes, Myint et al. [33] conducted a study to investigate the relationship between visual impairment and performance in 10 m air rifle competitions. The study included a sample of 10 athletes, each of whom underwent various visual function tests, such as visual acuity, contrast sensitivity, and visual field testing. The authors found that individuals with low vision had similar performance to athletes with better vision, suggesting that auditory information is more critical for aiming than the ability to see the target or screen. As a result, the changes made to para-shooting make the sport more equitable for people with visual impairments, supporting the idea that a single sporting class is sufficient to ensure fair competition.

Latham et al. [36] investigated the relationship between visual function and shooting performance in a sample of 23 (14 males, 9 females) visually impaired elite rifle shooters aged 49 ± 12 years (range 30–71 years). Scores were significantly higher for each shot in the prone position (Cohen’s *d* effect size of 1.66) than in the standing position. The scores did not differ according to the sex/gender of the athlete for either the prone or standing discipline. Furthermore, no significant relationship was found between shooting performance and its variability in the prone position and visual function status. Similar trends were observed for the standing position, with the only slight difference being that the relationship between peripheral visual function and shooting score was stronger in the standing position, although not statistically significant. Finally, the difference in standing shooting score and its variability between athletes with no peripheral visual function and those with some peripheral visual function was not significant, with a moderate effect size (*d* = −0.36). Overall, these data confirm the lack of evidence to support the need for more than one class in shooting with visual impairment and suggest possible compensatory mechanisms (e.g., postural stability induced by visual impairment may be compensated by increased reliance on somatosensory and/or vestibular information).

A study by Allen et al. [53] examined the effect of developmental history of visual impairment on shooting performance in a sample of 25 (16 males, 9 females) elite shooters with visual impairment aged 49 ± 11.6 years (range 15–66 years) to see whether the relationship was moderated by age at onset of impairment. Shooting scores did not differ by gender, but prone scores were significantly higher (Cohen’s *d* effect size of 1.81). No associations were found between shooting scores and distance visual acuity or contrast sensitivity in either the prone or standing competition. Furthermore, the scores did not depend on the type of vision loss (acquired versus congenital) or whether the athlete had residual vision.

Although the evidence presented suggests that only one sporting class is required for low-vision shooting competitions [33,36], it is critical to establish a reliable and legitimate threshold of impairment below which a competitor would be considered eligible to compete. This threshold should be an indication of the level of visual impairment that impairs performance in the absence of auditory guidance.

Allen et al. [6] conducted a study to address these knowledge gaps by analysing the level of visual impairment that might reduce shooting performance. The authors simulated six different levels of visual impairment in 19 able-bodied shooters and analysed the effect of simulated impairment (in terms of simultaneous reduction in visual acuity and contrast sensitivity) on their shooting performance. The results of the study showed a nonlinear relationship between simulated impairment levels and normalised scores. Specifically, shooting performance remained relatively unchanged in response to small reductions, but deteriorated more rapidly with larger reductions (less than 0.5 logMAR and 0.8 logCS). In addition, the visual function measures had a high ability to assess shooting performance, with 78% and 74% of performances classified appropriately using a logMAR cutoff of 0.53 and a logCS cutoff of 0.83, respectively. The shooting classification system currently used for athletes with visual impairment requires a minimum visual impairment of 1.0 logMAR. The authors believe that this is an appropriate criterion to exclude athletes with visual impairments from participation in sighted competitions. However, if the aim is to be more inclusive of people whose performance would be adversely affected by their vision, a more sensitive visual acuity cutoff would be required. Finally, the authors stress the importance of considering the effects of reduced contrast sensitivity when determining eligibility for visual impairment competitions.

In their study, Allen et al. [35] investigated how a reduction in visual acuity and/or contrast sensitivity visual acuity affected shooting performance. They used similar methods as the previous study but with a larger sample size (27 athletes with normal vision). These athletes were asked to shoot at a target wearing a range of filters and lenses that simulated varying degrees of visual acuity and contrast sensitivity loss. In this way, the authors were able to simulate the visual impairments of the athletes. To ensure the accuracy and reliability of the results, the authors used two different analysis techniques. The aim was to isolate and establish the independent effects of visual acuity and contrast sensitivity loss on shooting performance. After applying both techniques, the authors found that contrast sensitivity loss was a more reliable predictor of shooting performance than visual acuity loss. Specifically, statistical analysis (stepwise logistic regression models) showed that contrast sensitivity was the most important predictor of shooting performance, but visual acuity also contributed to the accuracy of the model. In other words, contrast sensitivity was the strongest predictor, but visual acuity also had a smaller but still significant effect on shooting performance. In another type of statistical analysis (decision tree analysis), contrast sensitivity was the only factor necessary to predict whether an athlete would have a shooting score below the predicted level. This analysis showed that contrast sensitivity was a very strong predictor of shooting performance and that it was even possible to predict shooting performance with a high degree of accuracy using only contrast sensitivity as a criterion.

Allen et al. [37] evaluated the feasibility and perceived benefits of a proposed new classification system that would consider both visual acuity and contrast sensitivity in determining an athlete’s class in para-shooting. The study involved 17 athletes with visual impairments, seven coaches, and four classifiers involved in para-shooting. Participants were asked to complete a survey to assess their perceptions of the proposed new classification system. The survey included questions about the understanding, feasibility, fairness, and potential benefits of the new system. The results of the study showed that athletes were unaware of the changes to the classification system due to poor communication, highlighting the need for better understanding among classifiers. Despite this, most participants were positive about the proposed new system. They believed it would improve the accuracy and fairness of assessing an athlete’s ability in sport by considering visual acuity and contrast sensitivity. In addition, participants anticipated several benefits from the new system, including increased transparency and consistency in classification procedures, reduced likelihood of misclassification, and increased fairness in competition.

Considering the studies mentioned above, it appears that the classification system centred around a functional class is accurate, and the minimum threshold for qualifying for a minimal impairment is precise. In the past, there has been concern about the absence of contrast sensitivity, but this latest assessment was incorporated into the new classification system and improved it.

### 4.3. Development of New Methods and Technologies in the Disciplines of Adaptive Shooting

The fact that few manufacturers currently develop adaptive sportswear specifically for athletes with disabilities highlights a significant gap in the sports industry. Despite the growing number of athletes with disabilities participating in sports, the availability of specialised adaptive sportswear is limited. This poses a significant challenge for para-athletes who may struggle to find clothing and equipment that meet their specific physical needs [61].

Hobbs-Murphy et al. [22] analysed the process and results of designing a jacket to improve the performance of para-shooters. The design process is described in detail, starting with the initial research phase, which involved observing and interviewing a para-shooter to learn about their needs and preferences. The study describes how the designers used this information to create prototypes of the shooting jacket, which were tested and refined through feedback from the athletes. The study also explains the key features of the shooting jacket, which include adjustable straps, a customisable back panel, and a removable front panel. These features were designed to provide comfort, support, stability, and improved posture. 

Laser targets have revolutionised shooting at the Paralympics, making it more accessible to and inclusive of athletes with disabilities. Instead of traditional paper targets, laser targets use an array of sensors and lights to track and visualise the shots fired by the shooter.

Abedini et al. [23] investigated the development of a controller that can be used in laser targets for para-shooting. The aim is to create a system that can accurately detect and record the position of shots fired by athletes, which is crucial for scoring purposes. The controller is based on a microcontroller programmed to detect the position of the shot fired using sensors and algorithms. The study illustrates the various stages of development and testing of the controller, including the design of the hardware and software, and the calibration and testing of the system. The test results showed that the controller was able to accurately detect and record the position of shots fired by athletes with a high degree of accuracy and consistency.

The case study by Jamara et al. [25] concerns a marksman who has visual field loss and diplopia, which affect his ability to perform well in target shooting. To address these problems, the shooter underwent low vision rehabilitation, which is a process of restoring or improving visual function in people with visual impairments. The rehabilitation programme included various interventions such as the use of magnification devices, contrast enhancement techniques, and prism glasses. Magnification devices, such as telescopic lenses, were used to improve the shooter’s visual acuity and enlarge the target. Contrast enhancement techniques, such as the use of brightly coloured targets or filters, helped the shooter to distinguish the target from the background. Prism glasses were used to reduce the effects of double vision by aligning the images seen by each eye.

Non-negative matrix factorisation (NNMF) is a technique for assessing muscle synergies, i.e., groups of muscles working together to perform a particular movement or task, by analysing surface electromyography [57,62]. 

Vendrame et al. [24] used electromyographic signal analysis methods to assess the activity of five muscles in a para-archer (sport class W1) and an able-bodied athlete. The authors identified three muscle synergies for both athletes; however, they observed differences in muscle synergy vectors (i.e., the relative weight of each muscle within each synergy) and synergistic activation coefficients (i.e., the relative contribution of the muscle synergy to the overall muscle activity pattern). These differences indicate that the two archers used different strategies during the different phases of the shooting action. In particular, the contribution of the biceps muscle rather than the posterior deltoid muscle during arrow extraction and aiming was more pronounced in the disabled athlete compared to his able-bodied counterpart.

The common theme of the studies presented is the importance of collaboration between athletes and designers in the development of adaptive sports equipment that can improve the performance and quality of life of para-athletes and provide ongoing support and follow-up to ensure their success. In addition, the accurate identification and analysis of the unique muscle activation patterns and strategies associated with specific conditions provide valuable benefits in the athletic preparation of athletes.

### 4.4. Biopsychosocial Aspects in Adaptive Shooting Disciplines

The social model of disability represents an evolution in the concept of disability, which is no longer seen as an individual and intrinsic condition of the person but rather as the product of social and environmental barriers that prevent persons with disabilities from participating fully in society [29,63]. These barriers can be physical, architectural, communicative, cultural, and attitudinal and can limit access to resources, opportunities, and choices available to people with disabilities. The social model of disability also emphasises the importance of removing these barriers and creating more inclusive and accessible environments for people with disabilities to enable them to participate actively in the social, economic, and cultural life of society [64]. This involves promoting policies, practices, and services that consider the needs and perspectives of people with disabilities, and creating a culture of inclusion that recognises and values the diversity and plurality of human experience [65,66].

Afacan Meltem Işık and Afacan Ersin [27] explored the visibility of disability in the context of para-shooting using the social model of disability. The study used a mixed methods approach to explore the experiences of 11 athletes, including the challenges they faced and the strategies they used to overcome them. The findings showed that although the social model provides a useful framework for understanding disability, it does not fully capture the complexity of the athletes’ experiences. The study also highlighted the importance of creating an inclusive and supportive environment for athletes with disabilities, which can help reduce the visibility of disability and promote participation and success. This includes providing appropriate accommodation and accessibility, as well as addressing negative attitudes and stereotypes towards people with disabilities.

The cross-sectional study conducted by Arkin and Budak [2] aimed to compare anthropometric characteristics, trunk stabilisation, body balance, body perception, and quality of life in 10 physically disabled professionals and 10 able-bodied archers using a variety of tests and questionnaires. The authors did not observe clear differences between groups in body perception and trunk stabilisation. As far as the anthropometric parameters are concerned, only a greater arm circumference by the para-athletes was found (*p* = 0.27 and *p* = 0.03 for the right and left arms, respectively). Interestingly, despite this physical advantage, para-athletes did not report greater levels of strength in the upper limbs. Furthermore, the latter had significantly lower levels of body balance (*p* < 0.05) and quality of life (*p* < 0.05) than their able-bodied counterparts. The absence of significant differences in some parameters studied may suggest a significant positive impact of sport on the psychophysical well-being of people with disabilities.

In their retrospective case report, Larsen et al. [30] examined a 23-year-old para-archer affected by McCune Albright syndrome, a rare genetic disorder affecting bones, skin, and hormone-producing glands. The report provides a detailed medical history, outlining the diagnosis and treatment of the archer’s syndrome, as well as her athletic career, training and competition schedules, and notable achievements in the sport of archery. Despite the challenges posed by her condition, the archer was able to excel in her sport, winning a bronze medal at the 2008 Paralympic Games in Beijing, China. The report also examines the archer’s disability status, measured through the completion of the Medical Outcome Survey Short Form-36 and the Hip Dysfunction and Osteoarthritis Score (HOOS). Compared to normative values for the US healthy female national age range (18–24 years) and National Collegiate Athletic Association (NCAA) athletes, the para-athlete scored higher on the subscales of general health, vitality, emotional role, and mental health. However, she scored lower than the comparison groups on the subscales of physical functioning, physical role, and body pain, indicating the impact of severe bilateral hip joint degeneration and leg length discrepancy on her mobility. Despite these challenges, the archer reported a higher perceived quality of life than the average patient with severe unilateral hip osteoarthritis, demonstrating her resilience and perseverance in the face of adversity. The report underscores the difficulties faced by athletes with disabilities, particularly those with rare conditions like McCune Albright syndrome while highlighting the critical role of medical teams and coaches in supporting their success. 

### 4.5. Kinematic and Biomechanical Assessment in Adaptive Shooting Disciplines

In para-archery, kinematic assessment is particularly important due to the unique challenges faced by disabled athletes [67]. Depending on the type of disability, an athlete may need to modify their technique to accommodate their physical limitations. 

Kim et al. [7] examined the kinematic characteristics of the upper body during shooting in seven para-archery athletes (three in the paralympic categories of ARST standing and four in ARW2 wheelchair archery). The participants had to make six shots at a total distance of 20 m, and the upper body kinematics were recorded using a 3D motion analysis system. The authors found that the ARW2 group required additional time during the draw and hold phases, 1.24 and 1.37 s longer than the ARST group, respectively. However, the ARST group took longer than the ARW2 group during the release and follow-through phases, although this difference was not significant. Furthermore, the change in the centre of mass trajectory during the pulling phase showed a greater degree of change in the direction of the anteroposterior axis in the ARW2 group than in the ARST group. Finally, there was no significant difference between the two groups in terms of the change in the centre of motion trajectory.

### 4.6. Epidemiology of Injuries in Adaptive Shooting Disciplines

Derman et al. [20] studied a sample of 139 para-archers (60 females, 79 males) and 154 para-shooters (54 females, 100 males), aged 12–75 years (mostly aged 35–75 years), who took part in the Tokyo 2020 Paralympic Games held during the COVID-19 pandemic. Athletes were prospectively monitored for a 15-day period, including the 3-day pre-competition period and the 12-day competition period. The incidence rate of illnesses for para-shooting was computed at 6.1 [95%CI 3.4 to 10.7] illnesses/1000 athlete days, one of the highest rates among 23 para-sports disciplines, after wheelchair tennis and before badminton. Fourteen illnesses were reported, 5.0% of all illnesses reported by all para-athletes during the study period, affecting 12 (7.8%) of the para-shooters competing. The incidence rate of illnesses for para-archery was 4.8 [95%CI 2.6 to 8.7] illnesses/1000 athlete days. Ten illnesses were reported, 3.6% of all illnesses reported, affecting 10 (7.2%) of the para-archers competing.

### 4.7. Bibliometrics-Based Analysis of Adaptive Shooting Disciplines Scientific Output

A few bibliometrics studies have investigated scholarly interest in paralympic disciplines. This scoping review was undertaken to fill this knowledge gap, showing that the community exploring para-sports and para-archery appears small, poorly connected, and highly heterogeneous, dispersed, and fragmented. In the field of sport, the creation of scientific networks and partnerships is crucial, especially when the topic (para-sport) is particularly complex. The present findings warrant the urgent need to conduct more, high-quality research in the field, by increasing the opportunities for networking, sharing knowledge, and, thus, advancing the scholarly discipline. 

### 4.8. Gaps in Knowledge and Future Directions for Adaptive Shooting Disciplines

Para-archery and para-shooting are two disciplines that have gained significant attention in recent years. Despite the growing popularity of these sports, there remain significant gaps in the literature. This section has a twofold objective: to identify the main gaps in the scientific literature regarding these two parasports, focusing on the key areas where research is lacking, and to suggest possible future directions for further study.

### 4.9. Biomechanics of Adaptive Shooting Disciplines

Research on the biomechanics of adaptive shooting is still limited. However, recent studies by Kim et al. [7] and Vendrame et al. [24] have shown significant biomechanical differences not only among different sports classes but also between para-athletes and able-bodied athletes. These results highlight the complexity of technical gestures and how they adapt to the specific characteristics of impairment. The use of new technologies and analysis techniques, such as the study of muscle synergies, promises to contribute to the understanding of the technical gesture and how the nervous system can coordinate and modulate complex movements [57,62]. This technology-supported research is crucial for the development of effective training programmes and the adaptation of equipment to the specific needs and safety of para-athletes [67].

### 4.10. Physio-Pathological Aspects in Adaptive Shooting Disciplines

Although it may appear to be a relatively static activity, adaptive shooting can still require significant physical effort and endurance, particularly in the upper body and core muscles [11]. However, there has been little research on the specific physical demands of adaptive shooting sports and how to train effectively for these demands. The study of physiological responses during physical activity plays a fundamental role in the world of sports and becomes even more relevant in the paralympic context. For example, it has been shown that para-athletes have different physiological responses depending on the type of disability. Athletes with upper motor neuron syndrome (a significant proportion of para-athletes are affected by this neurological syndrome) have been shown to experience an increase in spasticity and spastic dystonia during strenuous activity [18,68]. These conditions of muscular hypertonicity significantly alter performance, affecting the precision and effectiveness of movements and exponentially increasing the risk of musculoskeletal injuries [69].

### 4.11. Biopsychosocial Aspects in Adaptive Shooting Disciplines

There is a lack of research to aid in understanding the social and psychological impacts of adaptive shooting disciplines on athletes and the challenges they face in training and competition. It is widely recognised that individuals with disabilities tend to have a lower quality of life due to health issues and their basic conditions and may more frequently suffer from anxiety and depression compared to individuals without disabilities [70,71]. Although physical activity can help manage disability-related stress [72,73], athletes with disabilities and para-athletes face unique challenges, such as discrimination, lack of coaching professionalism, limited physical access, communication and economic barriers, and stigmatising situations [74]. To understand how these factors affect performance and develop strategies to improve mental alertness, concentration, and overall psychological well-being, further research is essential.

### 4.12. Training Methods and Programs for Adaptive Shooting Disciplines

No studies have investigated the training methods and programmes used by adaptive shooting athletes. The athletic preparation of a para-athlete requires a precise balance of load parameters, based on the specific needs related to the athlete’s condition. For example, the intensity of the training volume must be balanced with adequate recovery, which is usually longer than for able-bodied athletes [18]. However, para-athletes are often trained with similar load parameters to those of able-bodied athletes, which increases the risk of overtraining and injury [75]. Therefore, research in this area is crucial to improve the performance of para-athletes and to ensure that they receive adequate training and coaching.

### 4.13. Equipment Adaptations and Modifications for Adaptive Shooting Athletes 

Although some case studies have investigated equipment adaptations, such as the introduction of a bespoke jacket [22], the use of lasers to improve accuracy [23], and the use of magnification devices, contrast enhancement techniques, and prism glasses [25], there is a clear need for further research in this area. 

A large-scale analysis of the specific adaptations adopted by para-athletes and their impact on the use of new technologies is essential to demonstrate their effectiveness, safety, and fairness during competition. Furthermore, it is crucial to obtain more information on how the “Coronavirus Disease 2019” (COVID-19) pandemic is affecting adaptive shooting disciplines, as new potentially harmful infectious outbreaks may occur and hinder psychophysical preparations for the upcoming Paralympic Games in Paris 2024. Although there are recommendations for the use of equipment to maintain athletes’ physical conditioning, these strategies may not be applicable to adaptive shooting athletes due to their need for specific equipment and safe environments for training and competitions [76,77].

## 5. Conclusions

The available literature on adaptive shooting is still very limited, especially regarding para-archery as compared to para-shooting, highlighting the need for further research in many key areas to ensure a better understanding of the various disciplines and to provide adequate support to para-athletes. Such research is essential to promote the participation of a greater number of athletes with disabilities in adaptive shooting sports, improve their performance, develop effective training programs, and adjust equipment to enable them to achieve their sporting goals.

## Figures and Tables

**Table 1:** List of excluded studies and reasons.

Reference	Authors	Year	Reason for Exclusion
[42]	Bedir and Erhan	2021	Focusing on a different population of athletes
[43]	Betteridge	2010	Lack of sufficient quantitative data
[44]	Blecharz et al.	2014	Focusing on a different population of athletes
[45]	Duvall et al.	2021	Review study
[46]	Goosey-Tolfrey	2010	Focusing on a different population of athletes
[47]	Kim et al.	2019	Focusing on a different population of athletes
[48]	Marques and Alves	2021	Presenting data that could not be disaggregated and stratified by para-sport discipline
[49]	Petro et al.	2021	Focusing on a different population of athletes
[50]	Shin et al.	2011	Conference abstract
[51]	Yusuf	2021	Conference abstract
[52]	Zemková and Kováčiková	2023	Review study

**Table 2:** Main features of the studies included in the present scoping review. N/A = not available.

Study, Study Design	Journal, IF (2022), Web of Science/Clarivate Ranking, Subject Areas and Categories	Participant				Impairment	Sport	Main Aim	Main Finding
		Male	Female	Tot.	Age ± SD				
Physiological Responses in Adaptive Shooting Disciplines									
Castle et al. (2013) [8]; Prospective study	European Journal of Applied Physiology; 3.346; Physiology—SCIE (Q2); Sport Sciences—SCIE (Q2)	3	2	5	40.2 ± 1.8	3 spinal cord injuries 1 polio 1 spina bifida	Para-shooting	Assessment of the ability to acclimate to a hot environment	Partial acclimatisation with no response to sweating could be reported
Baumgart et al. (2018) [12]; Systematic review with meta-analysis and pooled-data analysis	Plos One; 3.752; Multidisciplinary Sciences—SCIE (Q2)	9	9	18	N/A	N/A	Para-shooting	Evaluation of VO_2peak_ values	Adaptive shooting sports discipline reported the lowest values compared to the other sports examined
		7	1	8	N/A	N/A	Para-archery		
Cooper et al. (1999) [13]; Crossover study	Assistive Technology; 2.17; Rehabilitation—SSCI (Q2)	2	2	4	44.8 ± 85	Paraplegia	Para-shooting	Evaluation of VO_2peak_	Absolute VO_2peak_: 1.9 ± 0.2 L∙min^−1^
		4	0	4	44.0 ± 11.3	Paraplegia	Para-archery		Absolute VO_2peak_: 1.5 ± 0.2 L∙min^−1^
Gass and Camp (1979) [14]; Longitudinal study	Medicine & Science in Sports & Exercise; 6.289; Sport Sciences—SCIE (Q1)	2	0	2	41.9 ± 2.5	Paraplegia	Para-archery	Evaluation of VO_2peak_	Absolute VO_2peak:_ 1.6 ± 0.3 L∙min^−1^
Veeger et al. (1991) [15]; Cross-sectional study	Medicine & Science in Sports & Exercise; 6.289; Sport Sciences—SCIE (Q1)	4	0	4	37.0 ± 5.1	N/A	Para-shooting	Evaluation of VO_2peak_	Absolute VO_2peak_: 1.3 ± 0.3 L∙min^−1^
		1	0	1	47	N/A	Para-archery		Absolute VO_2peak_: 1.4 L∙min^−1^
Nakagawa et al. (2020) [9]; Clinical case study	NeuroImage-Clinical; 4.891; Neuroimaging—SCIE (Q2)	1	0	1	35	Amputated	Para-archery	Comparison of brain activity during contraction of the muscles of the right toe, ankle, knee, and hip joint muscles	In the para-athlete, activation of the primary motor cortex appeared to be more widely distributed. Additionally, the areas of the primary motor cortex that were stimulated produced motor-evoked potentials that were larger than those observed in the control group
		17	2	19	N/A	Able-bodied	Control group		
Coudereau et al. (2006) [54]; Case–control study	Laterality; 2.167 Psychology, Experimental—SSCI (Q3); Psychology, Multidisciplinary—SSCI (Q3)	N/A	N/A	10	42.5 ± N/A	Vision impairment	Para-archery	Study of the perception of space, using a tactile bisection task	In the counter group, no spatial deviation was observed, whereas in the archers’ group, pseudo-neglect was present
		N/A	N/A	10	40.7 ± N/A	Vision impairment	Control group		
Bednarczuk et al. (2019) [16]; Repeated measures design	Journal of Sports Medicine and Physical Fitness; 1.669; Sport Sciences—SCIE (Q4);	N/A	N/A	14	37.9 ± 8.1	Visual impairment (starting class: 16 B1, 28 B2, and 13 B3)	Para-shooting	Comparison of static balance using different tests	Better balance on the left leg when the eyes are open than when they are closed was found. There were no differences in the level of dysfunction and disciplines evaluated
		N/A	N/A	16	34.9 ± 7.3		Para-cycling		
		N/A	N/A	8	23.2 ± 4.5		Football		
		N/A	N/A	19	25.7 ± 7.8		Goalball		
Kim and Woo (2013) [17]; Case–control study	Perceptual and Motor Skills; 2.212 Psychology, Experimental—SSCI (Q3)	N/A	N/A	12	40.8 ± 7.0	Spinal cord injury	Para-shooting	Comparison of brain activity during visuomotor tasks	Despite similar visuospatial brain activity in both groups, para-athletes exhibited a higher attentional demand during the aiming period
		N/A	N/A	12	19.6 ± 1.7	Able-bodied	Control group		
**Research Design and Measurement Methods for Evidence-Based Classification in Adaptive Shooting Disciplines**									
Myint et al. (2016) [33]; Cross-sectional study	*BMJ Open Sport & Exercise Medicine*; 0.81; Sport Sciences—ESCI (N/A)	7	3	10	45.0 ± 12.4	Visual impairment	Para-shooting	Assessment of the correlation between visual impairment and performance	No correlation between visual function and shooting performance could be detected
Allen et al. (2016) [6]; Cross-sectional study	*Frontiers in Psychology*; 4.232; Psychology, Multidisciplinary—SSCI (Q1)	9	10	19	21.0 ± 6.8	Able-bodied	Shooting	Determination of the level at which simulated visual impairment negatively impacts shooting performance	Mild reductions in visual impairment had no association with performance, while more significant reductions appeared to compromise performance
Allen et al. (2018) [35]; Cross-sectional study	*Frontiers in Psychology*; 4.232; Psychology, Multidisciplinary—SSCI (Q1)	14	13	27	26.9 ± 12.6	Able-bodied	Shooting	Assessment of the relative influence of simulating reduced visual acuity and contrast sensitivity to determine their effect on performance	Contrast sensibility was a better predictor of shooting performance than visual acuity
Latham et al. (2021) [36]; Cross-sectional study	*Journal of Sports Sciences*; 3.943; Sport Sciences—SCIE (Q2)	14	9	23	49.0 ± 1	Visual impairment	Para-shooting	Evaluation of the relationship between the degree of visual field function and performance	Visual field function was not associated with performance
Allen et al. (2019) [53]; Cross-sectional study	*Frontiers in Psychology*; 4.232; Psychology, Multidisciplinary—SSCI (Q1)	16	9	25	49.0 ± 11.6	Visual impairment	Para-shooting	Assessment of the correlation between visual impairment and performance	Performance was not influenced by the level of visual impairment in terms of visual acuity and contrast sensitivity
Allen et al. (2021) [37]; Cross-sectional study	*Journal of Sports Sciences*; 3.943; Sport Sciences—SCIE (Q2)	12	5	17	43.8 ± 12.3	Visual impairment	Para-shooting	Evaluation of opinions and perspectives on the recently proposed classification system	The classification system was deemed valid, but it raised several uncertainties. Additionally, useful advice was provided to enhance the implementation of future classification systems
		N/A	N/A	7	N/A	Able-bodied	Coaches		
		3	1	4	N/A	Able-bodied	Classifiers		
**Development of New Methods and Technologies** **in Adaptive Shooting Disciplines**									
Hobbs-Murphy et al. (2022) [22]; Case study	*Clothing and Textiles Research Journal*; 0.985; Social Sciences, Interdisciplinary—SSCI (Q4); Business—SSCI (Q4)	0	1	1	23	N/A	Para-shooting	Identification of the specific needs of the para-athlete and design of a jacket that can accommodate those needs	The jacket not only proved to be functional but also comfortable and highly effective
Abedini et al. (2019) [23]; Cross-sectional study	*Journal of Artificial Intelligence in Electrical Engineering*; N/A	N/A	N/A	N/A	N/A	N/A	Para-shooting	Design of a controller for laser targets	The designed controller can provide stable and accurate targets that respond quickly to the shooting action of the athlete
Vendrame et al. (2021) [24]; Case–control study	43^rd^ Annual International Conference of the IEEE Engineering in Medicine & Biology Society (EMBC)	1	0	1	34	Spastic paraparesis	Para-archery	Electromyographic analyses of 5 muscles to compare archery shooting techniques	Differences in motor strategies specific to shooting technique were identified between the two participants
		1	0	1	47	Able-bodied	Archery		
Jamara et al. (2008) [25]; Case study	*Optometry* N/A	1	0	1	67	Visual impairment	Para-shooting	Evaluation of rehabilitation techniques and devices	An improvement in visual function aimed at enhancing performance in target shooting was observed
**Biopsychosocial aspects in Adaptive Shooting Disciplines**									
Afacan M.I. and Afacan E., (2021) [27]; Qualitative study	*Journal of Educational Issues*; N/A	7	4	11	20–59	N/A	Para-shooting	Examination of the visibility of disability in terms of the social model	The visibility of disability is influenced by several factors such as classification system, media representation, and individual experiences
Larsen et al. (2010) [30]; Retrospective case report	*Disability and Rehabilitation*; 2.439; Rehabilitation—SSCI (Q2); Rehabilitation—SCIE (Q2)	0	1	1	22	McCune Albright syndrome	Para-archery	Documentation of the experiences and achievements of the athlete	The athlete was able to overcome adversity and excel in sport, demonstrating the importance of determination and resilience to achieve success
Arkin and Budak (2021) [2]; Cross-sectional study	*Sport Sciences for Health*; (N/A) Sport Sciences—ESCI (N/A)	4	6	10	35.4 ± 11.1	N/A	Para-archery	Examination and comparison of trunk stability, body perception, and general well-being	Able-bodied archers outperformed para-archers in reach tests, physical role difficulty, SF-36 physical function sub-scores, left horizontal adduction, and right internal rotation ROM, whereas para-archers demonstrated greater arm circumference
		6	4	10	34.2 ± 4.4	Able-bodied	Archery		
**Kinematic and Biomechanical Assessment in Adaptive Shooting Disciplines**									
Kim et al. (2021) [7]; Feasibility study	*International Journal of Environmental Research and Public Health*; 4.614; Public, Environmental, and Occupational Health—SSCI (Q1); Environmental Sciences—SCIE (Q2); Public, Environmental, and Occupational Health—SCIE (Q2)	3	1	4	46.5 ± 3.9	ARW2 (second wheelchair class—paraplegia or comparable disability)	Para-archery	Comparison and analysis of the kinematic characteristics of upper limb segments in seated and standing archers	ARW2 during the drawing phase took longer to completion compared to ARST. Additionally, ARW2 exhibited greater movement trajectory changes in the anteroposterior axis of their body centre during the drawing phase than ARST
		1	2	3	48 ± 2.6	ARST (standing archery class—loss of 25 points in the upper limbs or lower limbs)	Para-archery		
**Epidemiology of Injuries in Adaptive Shooting Disciplines**									
Derman et al. (2023) [20]; Prospective cohort study	*British Journal of Sports Medicine* 18.473 Sport Sciences—SCIE (Q1)	100	54	154	12–75	N/A	Para-shooting	Study of the disease incidence rate over a 15-day period, including the 3-day pre-competition period and the 12-day competition period	The incidence rate of illness for archery was one of the highest among the 23 disciplines (6.1 [95% CI 3.4 to 10.7] illnesses/1000 athlete days), while that of archers was slightly above average (4.8 [95%CI 2.6 to 8.7] illnesses/1000 athlete days)
		79	60	139	12–75	N/A	Para-archery		
		2371	1739	4110	12–75	N/A	21 different para-sports		

**Table 3:** The four most productive authors on adaptive shooting disciplines.

Author	Country	Number of Documents (%)	Links	Link Strength	Author Cluster	Publication Year Range
Allen, PM	UK	6	13	28	2	2016–2019
Mann, DL	The Netherlands	6	13	28	2	2016–2021
Latham, K	UK	5	11	25	2	2016–2021
Myint, J	UK	5	11	25	2	2016–2021

**Table 4:** Topological features of the scholarly community investigating para-shooting and para-archery.

Topological Features	Value
Average number of neighbours	16
Network diameter	1
Network radius	1
Characteristic path length	1
Clustering coefficient	1
Network density	1
Network heterogeneity	0
Network centralisation	0
Connected components	18

## Data Availability

Being a scoping review of the literature, no new data were generated.

## References

[1] Chen Y.-T., Mordus D., De Luigi A.J. (2018). Shooting Sports (Archery, Air Rifle, Trapshooting). Adaptive Sports Medicine.

[2] Arkin I., Budak M. (2021). Trunk Stabilization, Body Balance, Body Perception, and Quality of Life in Professional Physically Disabled and Able-Bodied Archers. Sport Sci. Health.

[3] World Archery (2022). Para Archery Classifiers Handbook.

[4] World Shooting Para Sport (2019). Classification Rules and Regulations.

[5] Mondal A., Pal S., Majumdar R. (2011). Anthropometry and Physiological Profile of Indian Shooter. IJASS.

[6] Allen P.M., Latham K., Mann D.L., Ravensbergen R.H.J.C., Myint J. (2016). The Level of Vision Necessary for Competitive Performance in Rifle Shooting: Setting the Standards for Paralympic Shooting with Vision Impairment. Front. Psychol..

[7] Kim T.-W., Lee J.-W., Kang S.-K., Chae K.-Y., Choi S.-H., Song Y.-G. (2021). A Feasibility Study of Kinematic Characteristics on the Upper Body According to the Shooting of Elite Disabled Archery Athletes. Int. J. Environ. Res. Public Health.

[8] Castle P.C., Kularatne B.P., Brewer J., Mauger A.R., Austen R.A., Tuttle J.A., Sculthorpe N., Mackenzie R.W., Maxwell N.S., Webborn A.D.J. (2013). Partial Heat Acclimation of Athletes with Spinal Cord Lesion. Eur. J. Appl. Physiol..

[9] Nakagawa K., Takemi M., Nakanishi T., Sasaki A., Nakazawa K. (2020). Cortical Reorganization of Lower-Limb Motor Representations in an Elite Archery Athlete with Congenital Amputation of Both Arms. NeuroImage Clin..

[10] Ravella K.C., Hutchinson M.R., Rocha Piedade S., Neyret P., Espregueira-Mendes J., Cohen M., Hutchinson M.R. (2021). Shooting Sports and Archery. Specific Sports-Related Injuries.

[11] Leroyer P., Van Hoecke J., Helal J.N. (1993). Biomechanical Study of the Final Push-pull in Archery. J. Sports Sci..

[12] Baumgart J.K., Brurok B., Sandbakk Ø. (2018). Peak Oxygen Uptake in Paralympic Sitting Sports: A Systematic Literature Review, Meta- and Pooled-Data Analysis. PLoS ONE.

[13] Cooper R.A., O’Connor T.J., Robertson R.N., Langbein W.E., Baldini F.D. (1999). An Investigation of the Exercise Capacity of the Wheelchair Sports USA Team. Assist. Technol..

[14] Gass G.C., Camp E.M. (1979). Physiological characteristics of trained Australian paraplegic and tetraplegic subjects. Med. Sci. Sports Exerc..

[15] Veeger H.E., Hadj Yahmed M., van der Woude L.H., Charpentier P. (1991). Peak oxygen uptake and maximal power output of Olympic wheelchair-dependent athletes. Med. Sci. Sports Exerc..

[16] Bednarczuk G., Wiszomirska I., Rutkowska I., Skowroński W. (2019). Effects of Sport on Static Balance in Athletes with Visual Impairments. J. Sports Med. Phys. Fitness.

[17] Kim W., Woo M. (2013). An Electrocortical Comparison of Elite Shooters with and without Disability during Visuomotor Performance. Percept. Mot. Skills.

[18] Puce L., Marinelli L., Pierantozzi E., Mori L., Pallecchi I., Bonifazi M., Bove M., Franchini E., Trompetto C. (2018). Training Methods and Analysis of Races of a Top Level Paralympic Swimming Athlete. J. Exerc. Rehabil..

[19] Grossmann F., Flueck J.L., Perret C., Meeusen R., Roelands B. (2021). The Thermoregulatory and Thermal Responses of Individuals with a Spinal Cord Injury During Exercise, Acclimation and by Using Cooling Strategies—A Systematic Review. Front. Physiol..

[20] Derman W., Runciman P., Eken M., Boer P.-H., Blauwet C., Bogdos M., Idrisova G., Jordaan E., Kissick J., LeVan P. (2023). Incidence and Burden of Illness at the Tokyo 2020 Paralympic Games Held during the COVID-19 Pandemic: A Prospective Cohort Study of 66,045 Athlete Days. Br. J. Sports Med..

[21] Thompson W.R., Vanlandewijck Y.C. (2013). Science and the Paralympic Movement. Br. J. Sports Med..

[22] Hobbs-Murphy K., Morris K., Park J. (2022). A Case Study of Developing a Paralympic Shooting Jacket for Disabled Athletes. Cloth. Text. Res. J..

[23] Abedini D., Alipour Banaei H., Yasrebi B. (2019). Controller Designing for Use in Laser Targets in Paralympics Shooting. J. Artif. Intell. Electr. Eng..

[24] Vendrame E., Rum L., Belluscio V., Truppa L., Vannozzi G., Lazich A., Bergamini E., Mannini A. Muscle Synergies in Archery: An Explorative Study on Experienced Athletes with and without Physical Disability. Proceedings of the 2021 43rd Annual International Conference of the IEEE Engineering in Medicine & Biology Society (EMBC).

[25] Jamara R., Potaznick W., Matjucha I. (2008). Low Vision Rehabilitation for a Target-Shooting Marksman with Visual Field Loss and Diplopia. Optom. J. Am. Optom. Assoc..

[26] Buts C., Bois C.D., Heyndels B., Jegers M. (2013). Socioeconomic Determinants of Success at the Summer Paralympics. J. Sports Econom..

[27] Afacan M.I., Afacan E. (2021). The Visibility of the Disability in Terms of the Social Model: Para Shooting Sport Sample. JEI.

[28] Dehghansai N., Pinder R.A., Baker J. (2022). Talent Identification and Development in Paralympic Contexts: Current Challenges. Front. Sports Act. Living.

[29] Puce L., Okwen P.M., Yuh M.N., Akah Ndum Okwen G., Pambe Miong R.H., Kong J.D., Bragazzi N.L. (2023). Well-Being and Quality of Life in People with Disabilities Practicing Sports, Athletes with Disabilities, and Para-Athletes: Insights from a Critical Review of the Literature. Front. Psychol..

[30] Larsen N., Nyland J., Roberts C.S. (2010). USA Paralympic Team Archer with McCune Albright Syndrome: A Retrospective Case Report. Disabil. Rehabil..

[31] Mann D.L., Tweedy S.M., Jackson R.C., Vanlandewijck Y.C. (2021). Classifying the Evidence for Evidence-Based Classification in Paralympic Sport. J. Sports Sci..

[32] McNamee M., Parnell R., Vanlandewijck Y. (2021). Fairness, Technology and the Ethics of Paralympic Sport Classification. Eur. J. Sport Sci..

[33] Myint J., Latham K., Mann D., Gomersall P., Wilkins A.J., Allen P.M. (2016). The Relationship between Visual Function and Performance in Rifle Shooting for Athletes with Vision Impairment. BMJ Open Sport Exerc. Med..

[34] Tweedy S.M., Beckman E.M., Connick M.J. (2014). Paralympic Classification: Conceptual Basis, Current Methods, and Research Update. PM&R.

[35] Allen P.M., Ravensbergen R.H.J.C., Latham K., Rose A., Myint J., Mann D.L. (2018). Contrast Sensitivity Is a Significant Predictor of Performance in Rifle Shooting for Athletes with Vision Impairment. Front. Psychol..

[36] Latham K., Mann D.L., Dolan R., Myint J., Timmis M.A., Ryu D., Frisson S., Allen P.M. (2021). Do Visual Fields Need to Be Considered in Classification Criteria within Visually Impaired Shooting?. J. Sports Sci..

[37] Allen P.M., Mann D.L., van der Linde I., Beukes E.W. (2021). Perspectives of a New Sport-Specific Para Shooting Classification System for Athletes with Vision Impairment. J. Sports Sci..

[38] Westphaln K.K., Regoeczi W., Masotya M., Vazquez-Westphaln B., Lounsbury K., McDavid L., Lee H., Johnson J., Ronis S.D. (2021). From Arksey and O’Malley and Beyond: Customizations to Enhance a Team-Based, Mixed Approach to Scoping Review Methodology. MethodsX.

[39] Bradbury-Jones C., Aveyard H., Herber O.R., Isham L., Taylor J., O’Malley L. (2022). Scoping Reviews: The PAGER Framework for Improving the Quality of Reporting. Int. J. Soc. Res. Methodol..

[40] Puce L., Trabelsi K., Trompetto C., Mori L., Marinelli L., Currà A., Faelli E., Ferrando V., Okwen P., Kong J.D. (2022). A Bibliometrics-Enhanced, PAGER-Compliant Scoping Review of the Literature on Paralympic Powerlifting: Insights for Practices and Future Research. Healthcare.

[41] Puce L., Biz C., Trompetto C., Marinelli L., Currà A., Cavaggioni L., Formica M., Vecchi V., Cerchiaro M.C., Trabelsi K. (2023). A Scoping Review with Bibliometric Analysis of Para-Rowing: State of the Art and Future Directions. Healthcare.

[42] Bedir D., Erhan S.E. (2021). The Effect of Virtual Reality Technology on the Imagery Skills and Performance of Target-Based Sports Athletes. Front. Psychol..

[43] Betteridge P. (2010). Physiotherapist Classifiers Ensure Competitive Fair Play in Para-Archery. J. Orthop. Sports Phys. Ther..

[44] Blecharz J., Luszczynska A., Scholz U., Schwarzer R., Siekanska M., Cieslak R. (2014). Predicting Performance and Performance Satisfaction: Mindfulness and Beliefs about the Ability to Deal with Social Barriers in Sport. Anxiety Stress Coping.

[45] Duvall J., Satpute S., Cooper R., Cooper R.A. (2021). A Review of Adaptive Sport Opportunities for Power Wheelchair Users. Disabil. Rehabil. Assist. Technol..

[46] Goosey-Tolfrey V. (2010). Supporting the Paralympic Athlete: Focus on Wheeled Sports. Disabil. Rehabil..

[47] Kim J.-T., Kim S.-Y., Oh D.-W. (2019). An 8-Week Scapular Stabilization Exercise Program in an Elite Archer with Scapular Dyskinesis Presenting Joint Noise: A Case Report with One-Year Follow-Up. Physiother. Theory Pract..

[48] Marques M.P., Alves A.C.D.J. (2021). Investigating Environmental Factors and Paralympic Sports: An Analytical Study. Disabil. Rehabil. Assist. Technol..

[49] Petro B., Lénárt Á., Gaál Z.A., Kojouharova P., Kökény T., Ökrös C., Czigler I. (2021). Automatic Detection of Peripheral Stimuli in Shooters and Handball Players: An Event-Related Potential Study. Exp. Brain Res..

[50] Shin S., Nam D., Kim D., Lee M., Nam Y. (2011). Verification of Upper-Limb Cardiopulmonary Fitness Level and Relevancy between Measurement Methods in Arm Ergometer and Wheelchair Treadmill of Korean National Shooters with Disabilities. Med. Sci. Sports Exerc..

[51] Yusuf R.B. (2021). Cognitive Component in Paralympic Archery: Baseline Analysis of Concentration. Int. J. Sport Exerc. Psychol..

[52] Zemková E., Kováčiková Z. (2023). Sport-Specific Training Induced Adaptations in Postural Control and Their Relationship with Athletic Performance. Front. Hum. Neurosci..

[53] Allen P.M., Latham K., Ravensbergen R.H.J.C., Myint J., Mann D.L. (2019). Rifle Shooting for Athletes with Vision Impairment: Does One Class Fit All?. Front. Psychol..

[54] Coudereau J.P., Gueguen N., Pratte M., Sampaio E. (2006). Tactile Precision in Right-Handed Archery Experts with Visual Disabilities: A Pseudoneglect Effect?. Laterality.

[55] Krawczyk-Suszek M., Martowska B., Sapuła R. (2022). Analysis of the Stability of the Body in a Standing Position When Shooting at a Stationary Target―A Randomized Controlled Trial. Sensors.

[56] Voss P., Thomas M.E., Cisneros-Franco J.M., De Villers-Sidani É. (2017). Dynamic Brains and the Changing Rules of Neuroplasticity: Implications for Learning and Recovery. Front. Psychol..

[57] Ghislieri M., Agostini V., Knaflitz M. (2020). Muscle Synergies Extracted Using Principal Activations: Improvement of Robustness and Interpretability. IEEE Trans. Neural Syst. Rehabil. Eng..

[58] Friedrich T.E., Hunter P.V., Elias L.J. (2018). The Trajectory of Pseudoneglect in Adults: A Systematic Review. Neuropsychol. Rev..

[59] Gray O.J., McFarquhar M., Montaldi D. (2021). A Reassessment of the Pseudoneglect Effect: Attention Allocation Systems Are Selectively Engaged by Semantic and Spatial Processing. J. Exp. Psychol. Hum. Percept. Perform..

[60] Vinet A., Le Gallais D., Bouges S., Bernard P.-L., Poulain M., Varray A., Micallef J.-P. (2002). Prediction of VO2peak in Wheelchair-Dependent Athletes from the Adapted Léger and Boucher Test. Spinal Cord.

[61] Bragança S., Castellucci I., Gill S., Matthias P., Carvalho M., Arezes P. (2018). Insights on the Apparel Needs and Limitations for Athletes with Disabilities: The Design of Wheelchair Rugby Sports-Wear. Appl. Ergon..

[62] Taborri J., Agostini V., Artemiadis P.K., Ghislieri M., Jacobs D.A., Roh J., Rossi S. (2018). Feasibility of Muscle Synergy Outcomes in Clinics, Robotics, and Sports: A Systematic Review. Appl. Bionics Biomech..

[63] Anastasiou D., Kauffman J.M. (2013). The Social Model of Disability: Dichotomy between Impairment and Disability. J. Med. Philos..

[64] Shakespeare T., Watson N. (2001). The Social Model of Disability: An Outdated Ideology?. Research in Social Science and Disability.

[65] Puce L., Marinelli L., Mori L., Pallecchi I., Trompetto C. (2017). Protocol for the Study of Self-Perceived Psychological and Emotional Well-Being of Young Paralympic Athletes. Health Qual. Life Outcomes.

[66] Marin-Urquiza A., Ferreira J.P., Van Biesen D. (2018). Athletic Identity and Self-Esteem among Active and Retired Paralympic Athletes. Eur. J. Sport Sci..

[67] Curran S.A., Frossard L. (2012). Biomechanical Analyses of the Performance of Paralympians: From Foundation to Elite Level. Prosthetics Orthot. Int..

[68] Puce L., Bragazzi N.L., Currà A., Marinelli L., Mori L., Cotellessa F., Chamari K., Ponzano M., Samanipour M.H., Nikolaidis P.T. (2022). Not All Forms of Muscle Hypertonia Worsen with Fatigue: A Pilot Study in Para Swimmers. Front. Physiol..

[69] Maia A.C., Hogarth L., Burkett B., Payton C. (2021). Improving the Objectivity of the Current World Para Swimming Motor Coordination Test for Swimmers with Hypertonia, Ataxia and Athetosis Using Measures of Movement Smoothness, Rhythm and Accuracy. J. Sports Sci..

[70] Krahn G.L., Walker D.K., Correa-De-Araujo R. (2015). Persons with Disabilities as an Unrecognized Health Disparity Population. Am. J. Public Health.

[71] Tough H., Siegrist J., Fekete C. (2017). Erratum to: Social Relationships, Mental Health and Wellbeing in Physical Disability: A Systematic Review. BMC Public Health.

[72] Ascione A. (2018). Sports Program to Promote the Wellbeing of People with Disabilities. Acta Medica Mediterr..

[73] Kiuppis F. (2018). Inclusion in Sport: Disability and Participation. Sport Soc..

[74] Iezzoni L.I. (2009). Public Health Goals for Persons with Disabilities: Looking Ahead to 2020. Disabil. Health.

[75] Hirschmüller A., Fassbender K., Kubosch J., Leonhart R., Steffen K. (2021). Injury and Illness Surveillance in Elite Para Athletes: An Urgent Need for Suitable Illness Prevention Strategies. Am. J. Phys. Med. Rehabil..

[76] Puce L., Trabelsi K., Ammar A., Jabbour G., Marinelli L., Mori L., Kong J.D., Tsigalou C., Cotellessa F., Schenone C. (2022). A Tale of Two Stories: COVID-19 and Disability. A Critical Scoping Review of the Literature on the Effects of the Pandemic among Athletes with Disabilities and Para-Athletes. Front. Physiol..

[77] Hasson R., Sallis J.F., Coleman N., Kaushal N., Nocera V.G., Keith N. (2022). COVID-19: Implications for Physical Activity, Health Disparities, and Health Equity. Am. J. Lifestyle Med..

